# Whole genome sequencing of historical specimens from the world's largest fungal collection yields high‐quality assemblies

**DOI:** 10.1111/nph.70472

**Published:** 2025-08-18

**Authors:** Torda Varga, Roseina Woods, Frances Pitsillides, Rowena Hill, Alona Yu Biketova, Theo Llewellyn, Brandon J. P. Shaw, Emily Hodgson, Brigid Wong, Jasmine Le, Josepha Becker, Alexander J. Bradshaw, Seth L. E. Blake, Clementine Geeves, Quentin Levicky, Lottie Goodman, Ruben L. Mole, Sidney L. Reed, M. Carly Lin, Emily Read, Keenan Harris, Raquel Pino‐Bodas, László G. Nagy, Anna Bazzicalupo, Ester Gaya

**Affiliations:** ^1^ Royal Botanic Gardens Kew Richmond TW9 3DS UK; ^2^ Earlham Institute Norwich Norfolk NR4 7UZ UK; ^3^ Synthetic and Systems Biology Unit Institute of Biochemistry, HUN‐REN Biological Research Centre 6726 Szeged Hungary; ^4^ Department of Life Sciences Imperial College London, Silwood Park Campus Ascot Berkshire SL5 7PY UK; ^5^ Biology Department Clark University Worcester MA 01610 USA; ^6^ Departamento de Biología y Geología Física y Química Inorgánica, Universidad Rey Juan Carlos (URJC) Tulipán s/n 28933 Móstoles Spain; ^7^ Instituto de Investigación en Cambio Global (IICG‐URJC) Universidad Rey Juan Carlos Tulipán s/n 28933 Móstoles Spain

**Keywords:** DNA extraction, fungariomics, fungi, genome assembly, museomics, PCR, whole genome sequencing

## Abstract

- High‐throughput molecular studies of museum specimens (museomics) have great potential in biodiversity research, but fungal historical collections have scarcely been examined, leading to no comprehensive methodological assessments. Here we present a whole genome sequencing (WGS) project conducted at the Fungarium of the Royal Botanic Gardens, Kew.
- DNA was extracted from 2104 specimens collected between 1770 and 2023, and we found that the specimen age had the smallest effect, while DNA purification and taxonomic identity had the greatest effect on DNA yield. We barcoded 771 specimens, and WGS was conducted on a subset of 394 specimens that were empirically selected for in‐depth analysis.
- We developed an automated assembly pipeline, integrating 16 different approaches. Starting from 220 libraries (excluding lichenised specimens), we produced 3143 assemblies using these approaches and found that there is no universal assembly method that can provide good‐quality genomes for all; rather, different approaches should be used depending on the library size and the specimen's age.
- Producing high‐quality genomes from specimens over 100 yr old is possible by using customised DNA extraction protocols and applying a multimethod bioinformatic approach. Whole genomes from historical collections will enrich genomics resources, accelerating biodiversity and evolutionary research, amongst others.

## Introduction

Historical collections have been the basis for scientific advances since the nineteenth century, including the discovery of new species to phylogenetics and biogeography studies (Funk, 2018). Biodiversity and evolutionary studies tackling current global challenges, including biodiversity loss, have gained insight from organism‐based historical collections such as herbaria for plants and fungaria for fungi (Funk, 2018). Ancient DNA (aDNA) from historical specimens has been utilised in single‐locus sequencing, high‐throughput sequencing such as restriction site‐associated DNA sequencing, genome skimming, RNA‐seq or whole genome sequencing (WGS), depending on the scope and objective of the study or the condition of the specimens (Burrell *et al*., 2015; Dodsworth *et al*., 2019; Raxworthy & Smith, 2021; Canales *et al*., 2022). Preservation technique, specimen age or the target tissue can be limiting factors in acquiring a high‐quality DNA extract for aDNA sequencing. Despite the difficulties of working with historical specimens, recent developments in museomics have paved the way for generating genome‐scale data (Burrell *et al*., 2015; Dodsworth *et al*., 2019; Kistler *et al*., 2020; Fong *et al*., 2023), resulting in the ‘renaissance’ of museum collections (Burbano & Gutaker, 2023).

Fungal collections preserve the taxonomic, biogeographic and chemical diversity of the fungal kingdom in the form of specimens and their associated metadata; yet, genome‐scale molecular mining of these collections remains scarce. The most common way to preserve fungal specimens in historical fungal collections is as dried fruiting bodies, the macroscopic sexual reproductive structures of fungi. Millions of fruiting bodies are preserved in fungaria world‐wide, providing resources for taxonomic, biodiversity, ecological, and evolutionary studies (Andrew *et al*., 2018). A systematic DNA barcoding study estimated that *c*. 70% of the species stored in the Fungarium of the Royal Botanic Gardens, Kew, are not represented in the GenBank nucleotide database, indicating a huge unsampled diversity in fungaria (Brock *et al*., 2009).

Despite the exponential increase in sequencing since that study, it still highlights the fact that sequencing fungal collections and depositing these data in online repositories has great potential to reveal hidden fungal diversity and provide tools to better understand fungal biology. The most common approach to harness aDNA from fungarium specimens is through Sanger sequencing of one or more barcoding loci (Brock *et al*., 2009; Sohrabi *et al*., 2010; Osmundson *et al*., 2013). Recent studies have performed amplicon sequencing (Forin *et al*., 2018; Gueidan *et al*., 2019; Kistenich *et al*., 2019; Dal Forno *et al*., 2022; Gueidan & Li, 2022) and target capture enrichment sequencing (Grewe *et al*., 2020; Liimatainen *et al*., 2022), increasing the amount of molecular information from historical fungal specimens. Fungarium omics (i.e. fungariomics) studies have focused on the applicability of genomics (Staats *et al*., 2013) or improving fungal phylogenetics (Dentinger *et al*., 2016), with there being only a few examples where comparative genomics was carried out (Bradshaw *et al*., 2024; Dirks *et al*., 2025). The impact of fungariomics research greatly depends on the quality of genomes produced. For example, nonrigorous and shallow sequencing could provide incomplete genomic data, and conventional bioinformatic methods could result in insufficient historical genome assemblies with missing or partially assembled genes. Incomplete genomic information could lead to inconclusive species tree estimates (e.g. Xi *et al*., 2015), and comparative genomic analyses could be flawed by falsely missing homologous genes.

Advancing the frontiers of fungariomics requires detailed methodological revision, from sampling to genome assembly; yet, fungariomic method assessments are sporadic and not comprehensive. Studies have focused on a specific taxonomic group or discussed one or some steps throughout the fungariomics workflow (e.g. Smith *et al*., 2020; Dal Forno *et al*., 2022; Miller *et al*., 2022). For example, Brock *et al*. (2009) and Dentinger *et al*. (2010) discussed the performance of DNA extraction and PCR amplification from historical fungal collections. Osmundson *et al*. (2013) assessed the performance of PCR on 5000 fungarium specimens and found that age negatively correlates with PCR success. In comparison with Sanger sequencing, amplicon next‐generation sequencing showed improved performance in acquiring barcoding sequences from historical specimens (Forin *et al*., 2018; Gueidan *et al*., 2019; Kistenich *et al*., 2019; Dal Forno *et al*., 2022; Gueidan & Li, 2022; Miller *et al*., 2022), yet only genome‐wide methods may unlock the full potential of fungaria. Target capture methods prove useful in taxon‐focused research and have been used to enrich 75 single‐copy genes in 19 mushroom‐forming fungal species of the genus *Cortinarius* (Liimatainen *et al*., 2022), 250 genes for a lichen family (Grewe *et al*., 2020) and > 2000 genes of ant‐cultivated fungi (Schultz *et al*., 2024). WGS studies of mushroom‐forming fungi have applied different DNA extraction, library construction and assembly methods (Staats *et al*., 2013; Dentinger *et al*., 2016; Bradshaw *et al*., 2024; Tremble *et al*., 2024; Dirks *et al*., 2025) with variable success in assembling genomes, providing fragmented assemblies (N50: 0.5–60 kb) or many missing conserved genes (BUSCO%: 30–95%). Despite the rising number of fungariomic studies, there is no standardised guidance for DNA extraction, genome sequencing and assembly methods. Additionally, the relationship between genome sequencing success of historical fungal material and specimen age, taxonomy, type of tissue, specimen collection condition (e.g. climate) and methods is yet underexplored.

Here, we provide a methodological assessment of a large‐scale fungarium WGS project, discussing each step of the workflow and providing statistical support based on the DNA extraction of > 2000 specimens collected between 1770 and 2023. We found that DNA yield can highly depend on the extraction method used and the taxonomic identity of the specimens. By developing an automated genome assembly pipeline, we produced > 3000 assemblies of 220 specimens using various methods. The analysis of this dataset showed that there is no universal approach for genome assembly, and the choice of assembly method depends on the specimen's age and the sequenced library size.

## Materials and Methods

### Sampling

Fungarium material was sampled from the Fungarium of the Royal Botanic Gardens, Kew (KM). Where possible, a 1–2‐mm^3^ section of material was taken from the hymenium (i.e. spore‐bearing structure) while sampling substrate or host material was avoided as much as possible. Small sporocarps placed on a sterile plastic tray were sampled under the dissecting microscope using sterile tools and transferred to a 2‐ml Eppendorf tube. All tools were sterilised between sampling events and routinely UV‐treated to avoid introducing contamination at this step. To compare the sequencing results of our historical specimens to fresh materials, we included cultures and field collections in our study. Cultures were obtained from the CBS‐KNAW culture collection from the Westerdijk Fungal Biodiversity Institute (WI‐KNAW). For each culture, a section of mycelium from axenic cultures was transferred to either 2% malt extract media or nutrient liquid agar, grown at 25°C on an orbital shaker at 120 rpm for 1 wk. Mycelia from liquid agar were collected via vacuum filtration and frozen at −80°C. Material from culture was then pulverised with two sterile stainless steel beads in a 2‐ml Eppendorf using a Mixer Mill MM 400 (Retsch GmbH: Haan, Germany). Field‐collected specimens were dried in a food dehydrator and processed within a few days after collection, following the protocol of the fungarium materials described above.

### DNA extraction and PCR amplification

Physical homogenisation of samples was conducted using an automated high‐throughput tissue homogeniser and cell lyser (SPEX CertiPrep™ Pulverizer and Cell Lyser 2010) with steel beads at 100 ***g*** m for 1 min. Homogenisation was repeated until the sample was finely ground, with sterile silica powder added if necessary.

Six main DNA extraction protocols were used to compare and optimise methods specifically for historical fungal specimens. Protocols included three commercially available kits (GENECLEAN® Ancient DNA Kit, Qiagen Plant DNA Mini, and DNeasy Blood & Tissue Kits), and two CTAB (cetyltrimethylammonium bromide) and one PTB (*N*‐phenacylthiazolium bromide) lysis buffer‐based chloroform : isoamyl alcohol purification methods.

We followed the manufacturer's instructions for the commercially available kits. The GENECLEAN® for Ancient DNA Kit was specifically designed to extract historical DNA and contains guanidine thiocyanate, which can prevent DNA from binding to molecules and can be used to separate cellular debris (Boom *et al*., 1999). All commercially available kits were silica column‐based purification methods.

CTAB lysis coupled chloroform : isoamyl alcohol purification protocols followed the commonly used steps (Doyle & Doyle, 1987) with unmodified lysis (CTAB‐a) or with overnight lysis in a CTAB buffer containing 25 μl of proteinase K (CTAB‐db). Briefly, homogenised tissue was placed in 750 μl of CTAB buffer preheated to 65°C with 25 μl of proteinase K (CTAB‐db) and 3 μl of β‐mercaptoethanol. Tubes were then placed in a benchtop incubator shaker at 65°C (time varied depending on modifications as described above).

In the PTB lysis protocol, homogenised tissue was placed in 690 μl PTB preheated to 37°C and incubated overnight at this temperature.

Following the CTAB or PTB lysis, we performed chloroform : isoamyl alcohol purification. Briefly, 750 μl of 24 : 1, chloroform : isoamyl alcohol was added to the lysate and incubated in a shaker at room temperature for 1 h. After a 10‐min centrifugation at 8600 ***g*** (9000 rpm), the supernatant was transferred to a 1.5‐ml tube. A 2× volume of −20°C isopropanol was added, and the tubes were left at −20°C for 2 wk. After this, the tubes were centrifuged at 1000 ***g*** (3000 rpm) for 5 min; the liquid was poured off and 500 μl of 70% ethanol was added. The tubes were incubated at room temperature in a shaker at minimum speed for 1 h. After centrifuging at 1000 ***g*** (3000 rpm) for 3 min, this ethanol cleaning process was repeated if the pellet was dark. The remaining liquid was poured off for a final time, and the pellets were left in a clean fume hood to dry before adding 30–100 μl of MilliQ water or TE buffer.

DNA concentration was measured using a Quantus™ Fluorometer following the manufacturer's protocol. PCR amplification of the ITS1, ITS2 or the whole ITS region (ITS1 + 5.8S + ITS2) and, in a few cases, the LSU of the nuclear ribosomal RNA was carried out as previously described (Nagy *et al*., 2012; Biketova *et al*., 2022) using the ITS1F, ITS2, ITS3, ITS4, ITS4B, LROR and LR7 primers (Vilgalys & Hester, 1990; White *et al*., 1990; Gardes & Bruns, 1993; Hopple & Vilgalys, 1994).

### Fungarium data compilation

We compiled metadata to get a balanced sampling based on taxonomy, collection date and locality, and to subsequently analyse the effect of these variables on the DNA yield and genome assembly. Our primary source of metadata originated from the internal database of Kew Fungarium, but in the event of missing or outdated information, some fields required updating or an informed estimate. In cases where the collection year was not noted, we approximated the year based on information about the associated collectors or other resources when possible. The type specimens' names and accession numbers were searched in the Species and Index Fungorum databases (www.indexfungorum.org). If a match was found, the collection date stated in those databases was used; otherwise, publications linked to the specimen were checked, and the publication year served as an estimate when suitable. When no data were available, the collector's name and location were used to estimate the collection period, and if necessary, the collector's lifespan provided an upper limit. If the collection year could be narrowed down to a period, the median year would be assigned to the specimen. If only the birth and death of the collector were known, the year of the death was considered to avoid overestimating the age of specimens. Taxonomic classification of specimens was based on species names recorded in the Fungarium and Index Fungorum databases, prioritising Index Fungorum, except for specimens with barcoding sequences published in this study, for which we validated the current names using the relevant literature.

The Köppen–Geiger classification (Kottek *et al*., 2006), the most frequently used climate classification system, was applied to investigate the climate dependence of museomics methods. The five climate groups of the system (based on vegetation) distinguish between the equatorial zone (A), the arid zone (B), the warm temperate zone (C), the snow zone (D) and the polar zone (E). These climate classes provide a way to combine complex and diverse climatic factors into discrete categories. The equatorial zone contains regions with some of Earth's warmest climates, including tropical rainforests. The arid zone is driven more by precipitation than by temperature and includes different types of deserts, such as cold deserts at high latitudes in Asia, South America, or in the Arctic and Antarctic regions. The mid‐latitude zones C and D include temperate and continental climates. Finally, zone E includes the coldest climates, including tundra and areas covered by snow and ice throughout the year.

**Fig. 1:**
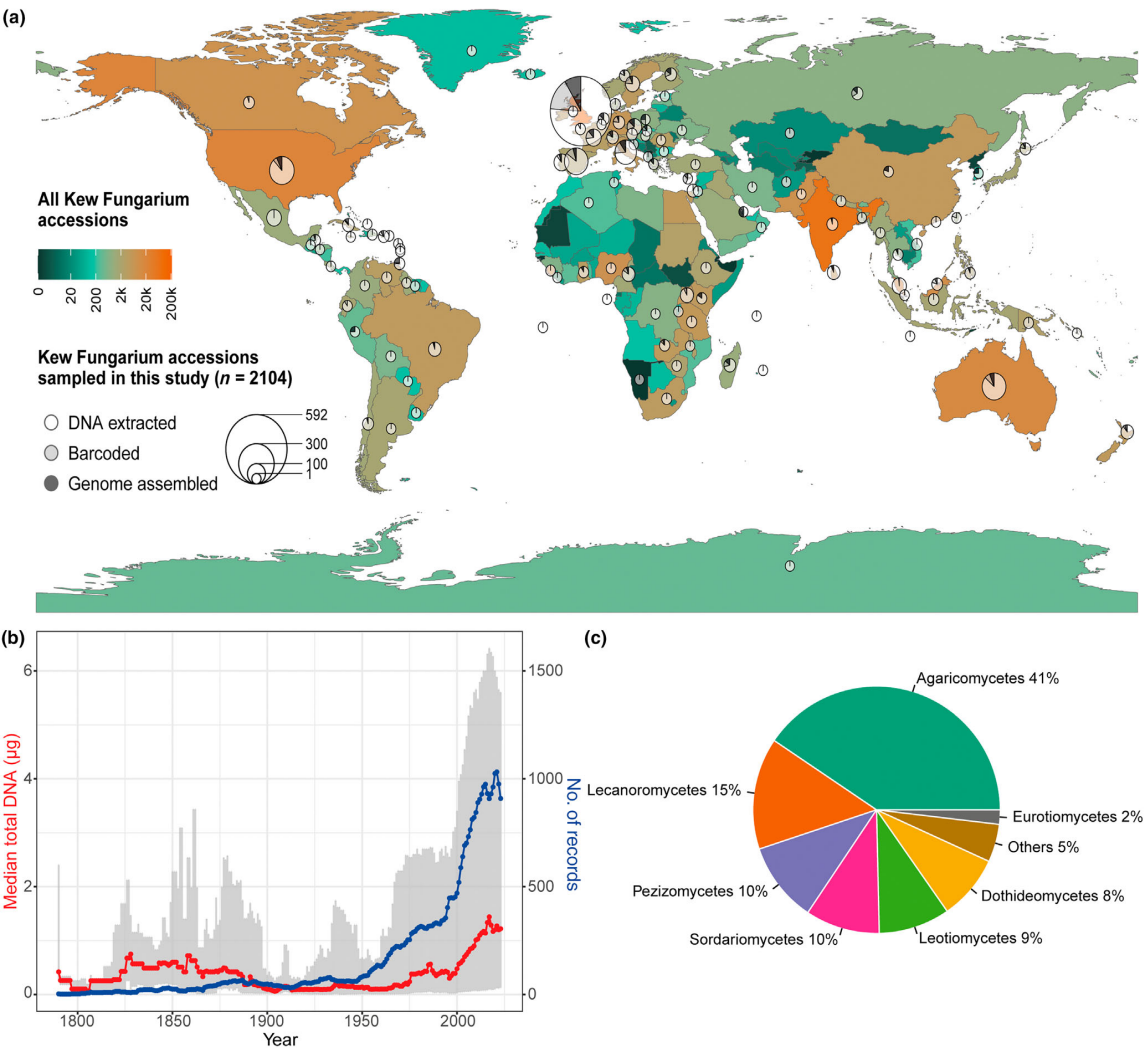
The spatial, temporal and taxonomic distribution of the 2104 fungarium specimens involved in this study. (a) Spatial heatmap of fungarium data. The heat colour represents the abundance of specimens (number of accessions) in the RBG, Kew fungarium summarised by country. The sampled (included in this study), barcoded and whole genome‐sequenced specimens are depicted by pie charts. (b) Temporal distribution of specimens from which DNA extraction was performed. The data were generated as a rolling median with a 20‐yr wide window and 1‐yr steps depicting median total DNA (left *y*‐axis) and number of extractions (right *y*‐axis). Bars represent the upper and lower quantiles of total DNA in each window. (c) The taxonomic distribution of the sampled specimens.

To assess spatial distribution, fungarium accessions were downloaded from the GBIF database (GBIF, 2024) on 11 October 2024. Total occurrences of 613 258 were filtered down to 547 747 that could be successfully matched to countries (or geounits in the case of noncontiguous territories) using ISO 3166‐1 alpha‐2 codes. We used the R packages rnaturalearth v.1.0.1 (Massicotte *et al*., 2023) and scatterpie v.0.2.4 (Yu, 2024) to visualise the global distribution of fungarium accessions and the number of accessions sampled, barcoded and WGS sequenced for each country or geounit (Fig. 1a). All mean values were reported as mean ± 1 SD. A complete list of all metadata collected is depicted in Supporting Information Table S1.

### Regression analysis on DNA yield

Starting with the 2524 extractions, we filtered data with < 15 observations per categorical variable (e.g. taxonomic classes) and excluded distorting and missing data (see below), which resulted in 1866 extractions suitable for a robust regression analysis. Regression analyses were carried out on two datasets. The input tissue dry weight was not measured in all cases; therefore, Dataset 2 (*n* = 1016) encompassed only samples where weight was recorded, whereas Dataset 1 included all samples (*n* = 1866). All datasets contained the total DNA (DNA_tot_) dependent variable and four explanatory variables (‘Age’, ‘Extraction protocol’, ‘Taxonomy’ and ‘Climate’) with Dataset 1 including the additional ‘Weight’ explanatory variable.

We calculated total DNA (DNA_tot_) by multiplying the concentration (ng μl^−1^) by the total volume (μl) of the extraction. Log_10_ transformation was performed on the raw data, and the ‘‐Inf’ values generated by 0 values (27 in Dataset 1 and 87 in Dataset 2) were replaced by one order of magnitude smaller value than the smallest logarithmic value. Continuous variables (total DNA and age) were scaled using the *scale* function of base R to be able to compare the individual effects of the explanatory variables. We chose a reference categorical level which has one of the lowest mean DNA_tot_, but a sufficient number of records (> 100). Accordingly, in all datasets, the ‘Qiagen Plant kit’ and the ‘*Dothideomycetes*’ were selected as the reference level in the ‘Extraction protocol’ and ‘Taxonomy’ variables, respectively. Before regression analysis, we removed samples from the dataset if they were represented by a category in the ‘Taxonomy’ or ‘Extraction protocol’ variable with < 15 records. We calculated the regression after removing distorting data points by calculating Cook's distance using the *cooks.distance* function of the base R package and removing data points with Cook's distance values three times higher than the mean. Multicollinearity was checked by calculating the generalised variance inflation factor using the vif function of the car v.3.1‐2 R package (Fox & Monette, 1992; Fox & Weisberg, 2018). Partial *R* ^2^ was calculated from the type II sum of squares by the *ANOVA* function of the car package to assess the relative importance of explanatory variables. All analyses were performed in R v.4.2.2 (R Core Team, 2021) and data handling and plots were made by using the tidyverse v.2.0.0 package (Wickham *et al*., 2019).

### Genome sequencing

DNA extractions with a sufficient amount of total DNA (> 1 or > 100 ng, depending on the library construction kit) were sent for WGS to Macrogen Europe Inc. The DNA integrity number (DIN) was measured by the company using TapeStation 4200. Library constructions were carried out based on the DNA quantity and level of fragmentation. Fragmentation was assessed based on the TapeStation's electropherogram (not DIN number), by assessing whether we could detect relatively abundant high molecular weight fragments or only short fragments (< 1 kb) were present. We used enzymatic fragmentation with Illumina Nextera XT DNA Kit (hereby ‘Nextera’; > 1 ng total amount of input DNA) or mechanical fragmentation with Illumina TruSeq DNA Nano Kit (hereby ‘TruSeq’; > 100 ng total amount of input DNA) if a large amount of high molecular weight DNA was present. If only low molecular weight (LMW) DNA could be detected, a modified version of the latter kit was used by skipping the fragmentation (hereby ‘TruSeq LMW’). The quality of the libraries was checked by an Agilent Technologies 2100 Bioanalyzer using a DNA 1000 chip. Paired‐read sequencing with 150 bp long reads was performed on an Illumina NovaSeq 6000 System or an Illumina HiSeq X System.

### Automated genome assembly pipeline and ITSx

We constructed an automated pipeline using a combination of shell and R scripts (https://github.com/vtorda/GenomeAssembler) and created a Docker image (https://hub.docker.com/repository/docker/vtorda/assembly_v0.0825/general) to efficiently assemble hundreds of short‐read libraries and test different assembly approaches. The pipeline uses two different read‐quality treatments (quality trimming or k‐mer‐based read correction), five different assemblers and different k‐mer parameters specific to a given assembler, outputting up to 16 different assemblies per library (Table S2). First, the adapters were removed using Trimmomatic v.0.40‐rc1 (Bolger *et al*., 2014); then quality trimming using Trimmomatic or read correction using Lighter v.1.1.2 (Song *et al*., 2014) was applied. The quality trimming was performed with the parameters of ILLUMINACLIP:{adapterfile}:2:30:10:2:true; LEADING:25; TRAILING:25; SLIDINGWINDOW:4:30 MINLEN:20. The Lighter read correction was carried out with a k‐mer length of 32 and a KmerGenie estimated genome size based on the adapter trimmed libraries. KmerGenie v.1.7051 (Chikhi & Medvedev, 2014) was used to estimate the genome size and an optimal k‐mer length for subsequent de Bruijn‐graph‐based assemblers. The assemblers Abyss v.2.3.7 (Jackman *et al*., 2017), Megahit v.1.2.9 (Li *et al*., 2015), SPAdes v.3.15.5 (Prjibelski *et al*., 2020), MaSuRCA v.4.1.0 (Zimin *et al*., 2013) and Idba‐ud v.1.1.3 (Peng *et al*., 2012) were used with various settings on the quality‐trimmed and read‐corrected libraries; thus, each assembly setting had at least two versions based on the read treatment. Fourteen assemblies were assembled by ABySS using seven k‐mers (33, 50, 70, 90, 110, 120 and a KmerGenie‐estimated kmer) and two k‐mer multiplicity threshold values (kc) of 2 and 3. For subsequent analysis, we chose a maximum of three assemblies per input library, one with the highest N50 value and two with the KmerGenie‐estimated k‐mer settings with kc = 2 and kc = 3. Libraries were assembled using Megahit with the –no‐mercy, ‐‐min‐count 3 and –k‐list 31, 51, 71, 91, 99 parameters, resulting in one assembly per input library. SPAdes also produced one assembly per input library by using the ‘only‐assembler’ mode without the genome polishing step because the reads had already been treated in two different ways, and polishing had a high computational resource demand. MaSuRCA was performed using the KmerGenie‐estimated genome size and a MaSuRCA‐ and a KmerGenie‐estimated k‐mer length, resulting in two assemblies per input library. Finally, IDBA‐UD was run with the ‐‐maxk 100, ‐‐min_contig 50 parameters, producing one assembly per library. At the end of the assembly step, a maximum of 16 draft, unfiltered assemblies were generated for subsequent quality measurements and comparison. The quality of the input read libraries and the assemblies was assessed by FastQC v.0.12.1 (Andrews, 2023), Kat v.2.4.1 (Mapleson *et al*., 2017), Samtools v.1.17 (Li *et al*., 2009), Quast v.5.2.0 (Mikheenko *et al*., 2018) and Busco v.5.4.7 (Manni *et al*., 2021). The quality measurements for various tools were summarised and organised by the MultiQC v.1.14 program (Ewels *et al*., 2016).

We extracted five barcoding nuclear rDNA loci (LSU, SSU, 5.8S, ITS1 and ITS2) from the draft assemblies using a hidden Markov model‐based searching program, the Itsx v.1.1.3 (Bengtsson‐Palme *et al*., 2013) with ‘–nhmmer T ‐‐save_regions all’ settings. Upon assessing the ITSx‐provided summary reports, we wanted to find out whether we could identify the expected taxon, based on rDNA sequences coming from the draft genomes. To do this, we performed a local search in an NCBI nucleotide database (created on 26 October 2024), specifically searching for fungal sequences. To perform thousands of searches in an efficient way, (1) we created a local database for only fungal sequences and (2) used the MMseqs2 v.16.747c6 search program (Steinegger & Söding, 2017). Subsequently, we used taxonkit v.0.20.0. with the ‘list –ids 4751’ argument to extract all taxon identifiers (IDs) linked to the Fungal kingdom. Then we downloaded the ‘accession to taxon’ NCBI database from the ftp://ftp.ncbi.nlm.nih.gov/pub/taxonomy/accession2taxid/nucl_gb.accession2taxid.gz address. We selected only fungal‐related accessions based on the taxon ID information and created a fasta formatted fungal database by using the *blastdbcmd* command of the Blast+ suite v.2.16.0 (Camacho *et al*., 2009). Then we set up an mmseqs query database from the ITSx extracted sequences longer than a locus‐specific value: 120 bp for 5.8S, ITS1 and ITS2; 500 bp for SSU and 600 bp for LSU. The query database and a search database from the filtered NCBI database were created by using the *mmseqs createdb* command. The mmseqs search was performed by setting the following parameters ‘‐‐search‐type 3’ and ‘‐s 7.5’. We assessed only hits with 98% identity and 10^−16^ E‐value. Taxonomic information was assigned to the target accessions by using the ‘accession to taxon’ NCBI database and the *taxonkit lineage* command.

Genome assemblies, ITSx and related analyses were carried out using the UKCropDiversity high‐performance computing (HPC) facility (Percival‐Alwyn *et al*., 2024).

### Regression analysis on the assembly quality

We measured the genome assembly quality as the percentage of complete conserved single‐copy orthologs found in the genome based on the fungal kingdom level orthoDBv10 database (consisting of 758 conserved orthologs) or as the contig size at 50% of the total assembly length (N50). The single copy ortholog percentage (BUSCO%) is a useful metric because it indicates how well ploidy is resolved and if fungal contamination could be present at the level of conserved genes. To analyse what affected BUSCO% or N50 values, we applied a linear mixed‐effects (LME) model that can account for the nested data structure. This nested nature of the data comes from the fact that the 16 assemblies with different conditions (fixed effects) were nested within specimens (random effects). Accordingly, we built LME models where BUSCO% or N50 was the dependent variable and the DNA_tot_, DIN value, climatic classes, age of the specimen, taxonomic identity at the level of classes, library preparation kit, the library size measured as the number of reads, the assembler program (ABySS, Megahit, SPAdes, MaSuRCA and IDBA‐UD) and read quality treatment (Trimmomatic or Lighter) were the explanatory variables. We performed the regression using the lmer function of the lme4 v.1.1‐35.5 package (Bates *et al*., 2015) after removing distorting variables by using Cook's distance and checked multicollinearity as above. To assess the individual importance of each explanatory variable, we calculated partial *R* ^2^ approximated by the Kenward–Roger approach (Edwards *et al*., 2008) using the r2beta function of the r2glmm v.0.1.2. package (Jaeger, 2016).

To understand what variables contributed to successful genome assemblies, we performed a multinomial logistic regression analysis using the *multinom* function of the nnet v.7.3‐19 package (Venables & Ripley, 2002) on a dataset where only the best assembly for each specimen was chosen based on BUSCO single‐copy gene percentage. Importantly, we analysed assemblies with 50% or higher single‐copy BUSCO% to exclude failed and poor assemblies. The assembler variable was set as an outcome, and the age, deduplicated library size, library concentration, climate, taxonomy, and read treatment were treated as the independent variables. First, we checked if the log odds have a linear relationship with independent variables and found that only the deduplicated library size needed to be scaled. Next, we determined which variable had a significant part of the model by performing model comparisons on the full model and models where one independent variable was removed. As a result, all subsequent analyses were performed on the following model: *assembler ~ library size + age + taxonomy + read treatment*. The optimal decay parameter was determined by performing a cross‐validation using the train function of the caret v.6.0‐94 package (Kuhn, 2008). To determine whether an independent variable had a significant effect on the probability that an assembly method outperforms others, marginal effects (ME, that is, trends) were estimated by using the avg_slopes function of the marginaleffects v.0.23.0 package (Arel‐Bundock *et al*., 2024). To disentangle the outcome of assembler choice along different predictors, we generated model‐based estimates (i.e. expected values) using the predict_response function of the ggeffects v.1.7.2 package (Lüdecke, 2018) by setting the nonfocal predictors to their means (margin = ‘marginalmeans’). To further explore the expected value sensitivity, we manually generated a fivefold repeated cross‐validation with 20 repeats and generated model estimates on each fold data.

## Results

### Large‐scale sampling and DNA extraction from Kew's Fungarium

We used Kew's Fungarium, containing *c*. 1.3 million specimens representing *c*. 49 000 species from all continents, to taxonomically and geographically evenly sample specimens for DNA extraction and WGS (Fig. 1a,c). Accordingly, specimens were sampled from all continents from the most speciose phyla, that is, *Basidiomycota* and *Ascomycota*. This sampling covers 409 families, of which 168 have never been WGSed and assembled (based on genome assemblies available in the JGI MycoCosm (Grigoriev *et al*., 2014) and NCBI databases (accessed on 22 April 2024)).

**Fig. 2:**
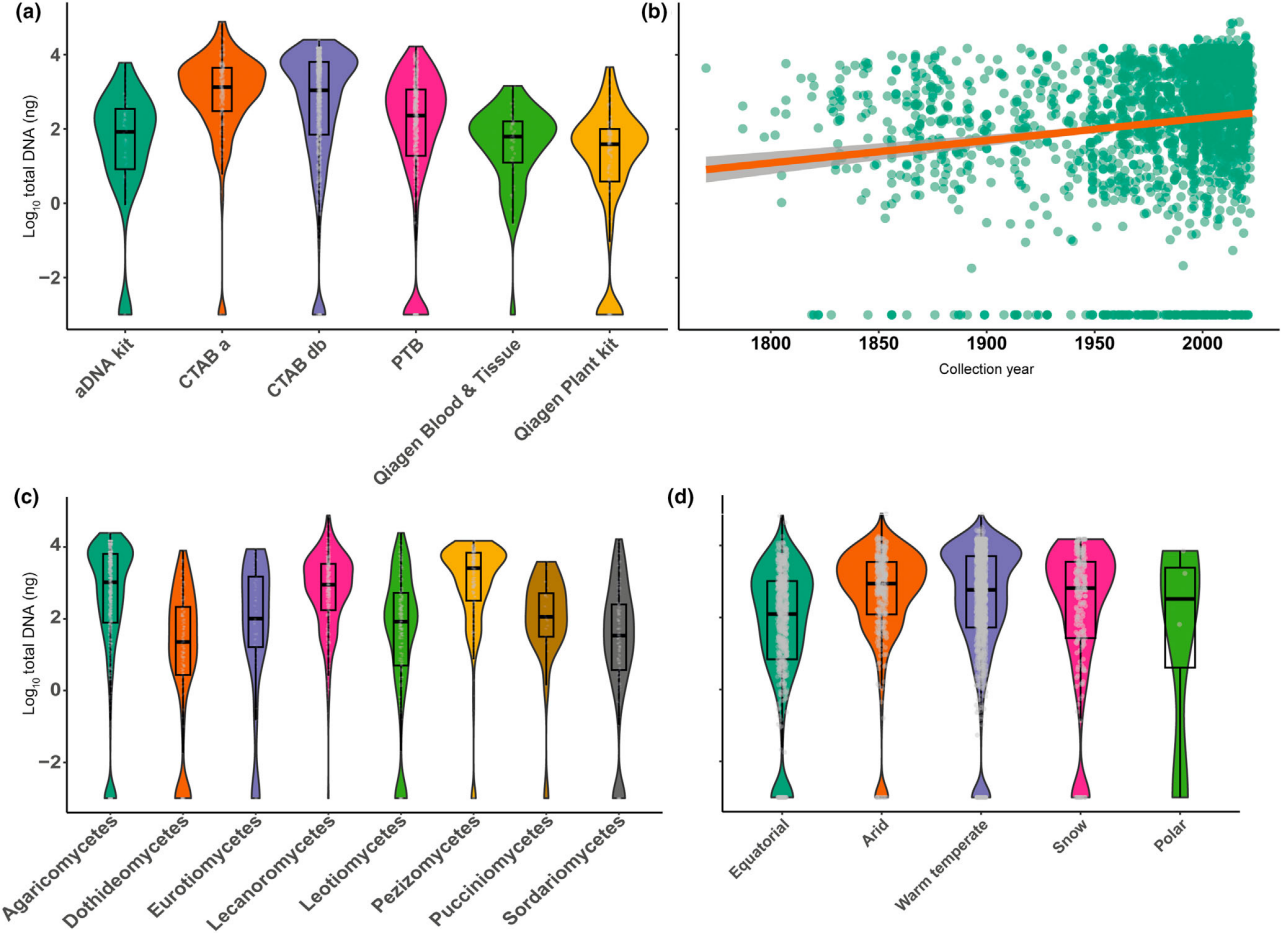
Assessing the DNA yield from historical fungal collections. (a) The variation between different DNA extraction methods. (b) The connection between DNA yield and collection year. (c) The variation across taxonomic classes. (d) The variation of DNA yield across different climatic classes. Boxplots and individual points within the violin plots represent quartiles and individual values respectively.

We performed 2524 DNA extractions from 2104 specimens collected between 1770 and 2023 (Fig. 1b; Table S1). We sampled specimens across a large collection period, including those older than 200 (*n* = 13), between 200–100 (*n* = 236) and 100–50 (*n* = 269) years. The remaining specimens (*n* = 1473) were 50 yr old or younger or had no age information (*n* = 113). DNA extraction was performed on an average of 14.99 ± 26.85 mg (min: 0.1 mg, max: 422 mg) of input tissue, resulting in 2.96 ± 5.75 μg total DNA (DNA_tot_) (min: 1.8 × 10^−5^ μg, max: 132 μg). A tendency for a higher DNA yield from specimens after the year 1975 can be observed (Fig. 1b), but DNA extraction can be affected by many factors (Fig. 2). For example, we used six main different DNA extraction methods and found a high variation in the DNA_tot_ between different protocols (mean DNA_tot_ = 0.003–4.96 μg). We included fresh field collections (*n* = 297) and mycelium cultures (*n* = 77) in our analyses as a reference that resulted in a higher mean total DNA (mean DNA_tot_ = 3.12 and 9.88 μg, respectively) relative to DNA extractions from fungarium samples (mean DNA_tot_ = 2.53 μg). Different taxonomic classes also showed variation (mean DNA_tot_ = 0.53–3.77 μg), and less variation was seen between Köppen–Geiger climate classes (mean DNA_tot_ = 1.31–3.42 μg). Interestingly, there was only a weak positive correlation between the input dry weight of the fungal tissue and the total DNA (Pearson's *R* ^2^ = 0.08, Fig. S1).

### Statistical assessment of the DNA yield from historical specimens

The yield of the DNA_tot_ extracted from historical fungal specimens could depend on many factors; thus, we performed a multivariate linear regression analysis with total DNA as a dependent variable to accommodate multiple effects of independent variables (Age, Extraction protocol, Taxonomy (Class), Climate and Weight). Starting with the 2524 extractions, we filtered out data with less than 15 observations per categorical variable (e.g. taxonomic classes) and excluded distorting and missing data that resulted in 1866 extractions. This (Dataset 1, *n* = 1866) did not include weight data in all cases; therefore, we analysed a subset where the weight variable was included (Dataset 2, *n* = 1016) (Tables 1, S3).

**Table 1:** Results of multivariate linear regression analyses on DNA yield.

Explanatory variables	Dataset 1 (*n* = 1866)					Dataset 2 (*n* = 1016)				
	df	*F* value	*P* value	Partial *R* ^2^	ΔAIC	df	*F* value	*P* value	Partial *R* ^2^	ΔAIC
Age	1	90.6	5.3 × 10^−21^	0.03	50.7	1	29.2	8.2 × 10^−8^	0.40	15.4
Extraction protocol	5	46.0	6.3 × 10^−45^	0.08	143.8	5	6.4	7.4 × 10^−6^	0.66	44.2
Taxonomy (Class)	7	41.9	3.4 × 10^−55^	0.13	245.7	6	13.9	2.8 × 10^−15^	0.75	63.9
Climate	3	20.3	5.8 × 10^−13^	0.03	54.6	3	8.8	9.4 × 10^−6^	0.50	20.5
Weight	–	–	–	–	–	1	14.3	1.6 × 10^−4^	0.15	2.7

We assessed two datasets with (Dataset 2) and without (Dataset 1) input tissue weight.

The regression analysis using Dataset 1 explained 27% of the variance in DNA_tot_ (*R* ^2^ = 0.27, *F*_(16,1866)_ = 42.2, *P* = 2.2 × 10^−16^). All variables were significant parts of the model, but with different levels of relative importance (Table 1). We found that taxonomy (*F*_(7,1866)_ = 41.9, *P* = 3.38 × 10^−55^) and the extraction protocol (*F*_(5,1866)_ = 46.0, *P* = 6.32 × 10^−45^) had the biggest effect on the variance with the highest partial *R* ^2^ values of 0.13 and 0.08, respectively, followed by age and climate (Table 1). A similar pattern was found in the case of Dataset 2, where the weight variable was included, but interestingly, the input dry weight had the smallest but positive significant effect on DNA_tot_ (*F*_(1,1016)_ = 14.3, *P* = 1.63 × 10^−4^). The weak explanatory power of input dry weight is also visible by the scattered distribution of weight values along DNA_tot_ with a small correlation coefficient (Pearson's *R* ^2^ = 0.08, Fig. S1). Because the weight variable had the most negligible effect on DNA_tot_ and model comparisons showed that the model without the weight variable (AIC = 2537) is comparable with the full model (AIC = 2539), we detail the individual variables based on Dataset 1 where more measurements are available.

The taxonomic classes had one of the biggest effects on the variance (Fig. 2c). The standardised coefficient for the *Pezizomycetes* was the highest (β = 0.78, *t* = 12.3, *P* = 2.85 × 10^−33^, DNA_tot_ = 3.84 ± 3.8 μg), followed by *Agaricomycetes* (β = 0.58, *t* = 10.6, *P* = 2.16 × 10^−25^, DNA_tot_ = 4.17 ± 5.2 μg) relative to *Dothideomycetes* (DNA_tot_ = 0.42 ± 1.0 μg). Other classes had significant but less positive effects (*Eurotiomycetes*, *Lecanoromycetes*, *Pucciniomycetes*) or no effect (*Sordariomycetes*) on the total DNA. The higher relative importance of the extraction protocol (*F*_(5,1866)_ = 46.0, *P* = 6.32 × 10^−45^, partial *R* ^2^ = 0.08) could be explained by the differences in precipitation‐based (CTAB, PTB) and silica column‐based methods (Fig. 2a). The CTAB methods (CTAB a: β = 0.91, *t* = 11.5, *P* = 1.86 × 10^−29^, DNA_tot_ = 5.60 ± 13.9 μg and CTAB db: β = 0.70, *t* = 11.0, *P* = 1.71 × 10^−27^, DNA_tot_ = 3.99 ± 5.0 μg) and the PTB method (β = 0.68, *t* = 10.1, *P* = 2.0 × 10^−23^, DNA_tot_ = 1.45 ± 2.5 μg) all performed well, positively affecting total DNA yield relative to the Qiagen Plant kit. These methods are based on a phenol–chloroform purification, while the other silica column‐based methods, such as the aDNA (DNA_tot_ = 0.44 ± 1.1 μg), Qiagen Plant (DNA_tot_ = 0.20 ± 0.7 μg) and Qiagen Blood & Tissue kits (DNA_tot_ = 1.80 ± 1.7 μg) resulted in a lower total DNA yield, suggesting a limit in the DNA binding capacity of the silica‐based column purification. The climatic classes and the age had the smallest relative contribution to the variance in total DNA, indicated by both having a partial *R* ^2^ value of 0.03 (Fig. 2b,d; Table 1). The analyses of the climatic classes revealed that specimens originating from arid (β = 0.20, *t* = 3.72, *P* = 2.07 × 10^−4^), warm temperate (β = 0.27, *t* = 7.41, *P* = 1.94 × 10^−13^) and snow climates (β = 0.32, *t* = 5.64, *P* = 1.98 × 10^−8^) had an increasing positive effect on the total DNA relative to the equatorial climate (Fig. 2d). Specimen age has one of the smallest effects on DNA yield (Fig. 2b, β = −0.14, *t* = −7.3, *P* = 4.9 × 10^−13^). Yet, the average DNA_tot_ was only 3.6 times lower among samples from the 19^th^ century (DNA_tot_ = 1.12 ± 2.32 μg) than among the specimens collected in the 21^st^ century (DNA_tot_ = 3.99 ± 7.33 μg), suggesting that a sufficient amount of DNA can be extracted from old specimens.

### DNA barcoding and library construction

We performed barcoding before WGS using Sanger sequencing of the nuclear ITS region to ensure that DNA from the target taxon had been successfully extracted. Accordingly, we performed 1686 PCR reactions and sequenced 771 specimens (Fig. 1a). Of the 771 sequences, 130 and 359 were contaminated and had poor quality, respectively. In total, 319 new barcoding sequences were published (BioProject accession no.: PRJNA1279621), covering 99 families and 153 genera (Table S4).

Next, we selected 722 extractions in multiple rounds to assess DNA size distribution and the level of fragmentation using a TapeStation device. The selection was based on empirical decisions based on judging the importance of a specimen (i.e. whether it is a type specimen or belongs to a higher level taxon with no genomic data). The mean DNA_tot_ of this selection was double compared to the extractions that were not sent to the company (Table S5), showing that we also favoured extractions with higher DNA yield. After measuring DNA integrity, we decided whether an extraction should be put forward to a library preparation step and which library preparation should be used from the three approaches. At this decision, we considered the importance of a specimen as above, but the DNA_tot_ was less important than the DNA fragmentation measured as a DIN value and the length of the most abundant fragment. We note that the DIN value was less informative than the visual inspection of the electropherograms, and the most abundant fragment size could not be measured if no ‘peak’ was detected in 427 cases out of the 722. Yet, we could describe some trends using DIN and fragment size statistics. The 442 extractions that were introduced to the library construction step had similar DNA_tot_ relative to extractions held, but they had a higher DIN value (3.2 vs 2.2 on average) and almost two times longer most abundant fragment sizes (16.9 kb vs 7.8 kb on average) (Table S5). We used enzymatic fragmentation with Illumina Nextera XT DNA Kit for extractions (*n* = 180, DIN_mean_ = 4.9) with low input DNA (DNA_tot_ > 1.2 ng) or mechanical fragmentation with Illumina TruSeq DNA Nano Kit for extractions (*n* = 98, DIN_mean_ = 3.4) with moderate input DNA (DNA_tot_ > 318 ng). The mechanical fragmentation was skipped for specimens with a low abundance of fragments in the high molecular weight region but a high abundance of fragments in the LMW region (Truseq LMW approach, *n* = 175, DIN_mean_ = 2.2) (Table S5). Out of the 442 libraries produced, we skipped 46 libraries due to their poor quality and sequenced 396 libraries with 13.07 ± 16.95 ng μl^−1^ of concentration and 455 ± 255 bp mean insert size.

### High‐quality draft genome assemblies from historical specimens obtained with an automated pipeline

To determine what affects the assembly quality measured by BUSCO single‐copy gene percentage (BUSCO%) and N50, we developed an automated assembly pipeline (see the Materials and Methods section) that produced up to 16 alternative assemblies starting from one paired‐end library (Table S2). We did not analyse lichen species that would complicate the comparison of metagenomes; thus, by excluding these, we produced 3143 assemblies from 220 paired libraries. The quality of genome assemblies varied across specimens and analyses. Considering only the best assembly for each specimen, the average single‐copy BUSCO%, N50, and genome size were 86 ± 23%, 40.7 ± 120 kb, and 61 ± 47 Mb, respectively (Table S6).

We found that these draft assemblies could have a low proportion of extrinsic DNA content. First, 159 out of 220 assemblies reached higher than 90% single‐copy BUSCO%, and the average duplicated BUSCO% was 4.3% (Fig. S2), which is a comparable value found in genome assemblies from fresh cultures. This result suggests a low level of contamination and that the haplotypes were collapsed successfully in most cases. Second, we extracted nuclear rDNA loci (5.8S, ITS1, ITS2, LSU, SSU) from the draft assemblies using ITSx to identify possible contamination (Table S7). ITSx detected 6.1 ± 14.4 number of rDNA sequences in 205 genomes out of the 220. However, a kingdom‐level taxon was assigned to only 104 genomes, out of which 86 had an rDNA sequence matching Fungi and 57 matching other taxa (‘Metazoa’, ‘Tracheophyta’, ‘Greenalgae’, ‘Liverworts’, ‘Oomycetes’, ‘Rhizaria’, ‘Amoebozoa’). We wondered if genomes with outstanding sizes (> 100 MBp based on contigs longer than 1 kbp) could be the result of contamination, but we could not find a correlation between genome size and the number of ITSx extracted rDNA (Pearson's *R* ^2^ = −0.02), that of Quast detected rDNA (Pearson's *R* ^2^ = 0.33) or the duplicated BUSCO% (Pearson's *R* ^2^ = 0.21). Contrarily, we found a high correlation between the genome size and the number of predicted genes longer than 300 bp (Pearson's *R* ^2^ = 0.73). The low duplicated BUSCO% is an indication that these genomes possibly went through a true expansion, but it requires further thorough investigation.

Third, upon the ITSx‐provided summaries, we were interested in whether the sequenced draft genomes could be identified as the source specimen based on rDNA sequences. For this, we searched the ITSx extracted sequences in an NCBI nucleotide database containing only fungi, and we found that 72% and 49% of the genomes matched with a sequence at the genus and species level, respectively (percentage identity > 98%, *E*‐value < 10^−16^).

**Fig. 3:**
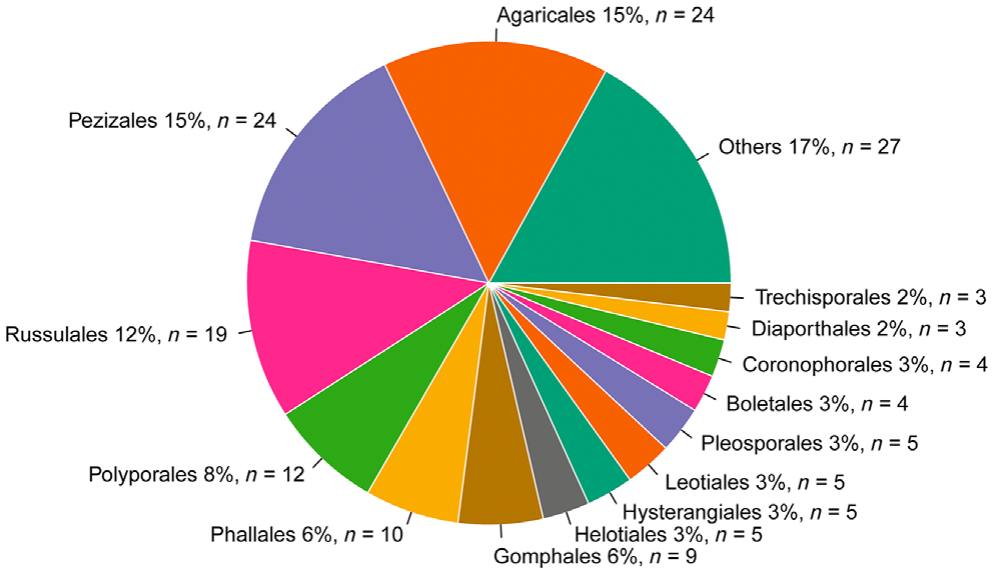
The proportion of orders with *de novo* high‐quality assemblies (single‐copy BUSCO% > 90) produced in this study.

Good‐quality draft assemblies (single‐copy BUSCO% > 90) were also obtained for many of the oldest specimens in our study, including 23 type specimens. For example, the assembly of a 180‐yr‐old holotype of a false truffle (*Hysterangium nephriticum*) resulted in a 63‐Mbp genome with 95% single‐copy BUSCO%, even though it had a relatively fragmented assembly with a N50 of 3.8 kb. These *de novo* high‐quality assemblies cover 31 orders with a limited number of published genomes, emphasizing the future contribution to potential upcoming genomic projects (Fig. 3; Table S6). For example, we assembled ten and four new genomes from the *Phallales* (*Agaricomycetes*, *Basidiomycota*) and *Coronophorales* (*Sordariomycetes*, *Ascomycota*) orders, with only four and three genomes deposited in the NCBI genome database (accessed on 05 January 2025).

### No single assembly method offers the best result for all, but library size, read treatment and taxonomy affect the choice of the assembler

**Fig. 4:**
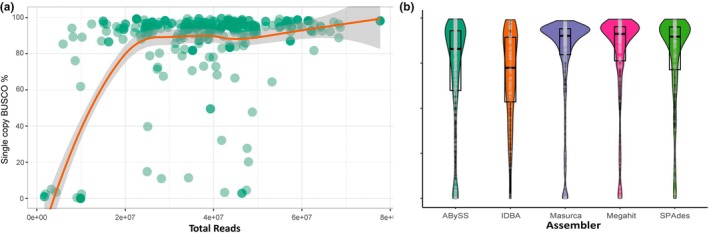
Quality of the assembled libraries based on single‐copy BUSCO percentage statistics. (a) The BUSCO% depends on the sequenced library size. (b) BUSCO% variation across different genome assembly methods. Boxplots and individual points within the violin plots represent quartiles and individual values respectively.

We found substantial variation amongst assembly approaches and different traits of the specimens. To understand what affects the quality of an assembly, the effect of library size (number of reads), the total DNA extracted, age, DIN value, library preparation kit, library concentration, climate, taxonomy (class), assembler (ABySS, IDBA, Masurca, Megahit, SPAdes) and read treatment (quality trimming or read correction) were analysed in a LME model (Table S8). We found that the fixed effects alone can explain 27% and 36% of the variance in BUSCO% and in N50, respectively. The largest portion of the BUSCO% variance was explained by the assembler (Partial *R* ^2^ = 0.30, *F*_(4,2121)_ = 228.1, *P* = 5.43 × 10^−163^) followed by the taxonomy (Partial *R* ^2^ = 0.12, *F*_(10,152)_ = 20, *P* = 3.35 × 10^−2^) and the library size (Partial *R* ^2^ = 0.07, *F*_(1,152)_ = 11.7, *P* = 7.98 × 10^−4^). The SPAdes, Megahit and Masurca assemblers had a significantly positive effect on the BUSCO% (Fig. 4b) with the Masurca having the biggest (β = 0.28, *t* = 21.70, *P* = 1.95 × 10^−94^), while IDBA had a negative significant effect relative to ABySS (β = −0.16, *t* = −8.63, *P* = 1.14 × 10^−17^). However, the relationships changed if we considered the simultaneous effect of taxonomy and assembler, introducing an interaction term between them (Fig. S3). The positive effect of Masurca and SPAdes was only present among *Pezizomycetes*, and ABySS showed significantly better results in other classes. The exception was *Agaricomycetes*, where no significant differences were found between ABySS, Masurca and Megahit. The positive effect of the library size (Fig. 4a, β = 0.21, *t* = 3.42, *P* = 7.98 × 10^−04^) could indicate that a higher portion of unique DNA fragments could be grasped with deep sequencing (> 40 M reads), facilitating the assembly process.

A smaller but significant portion of the variance in BUSCO% was explained by age (*R* ^2^ = 0.04) and climate (*R* ^2^ = 0.06), yet no significant differences were found between the climatic classes. Interestingly, the DIN, the library preparation kit, the extracted total DNA and the read treatment did not have a significant effect on the BUSCO%. All these variables except the read treatment affect the quality of the sequencing libraries; thus, the lack of significant effect of these variables could be attributed to the high quality of the sequencing libraries produced. Accordingly, the libraries reached high library concentration (69 ± 83 nM) with 311–700 bp insert size, excellent for Illumina sequencing.

The N50 variance is affected by only the assembler (Partial *R* ^2^ = 0.53, *F*_(4,2275)_ = 602.6, *P* = 1.00 × 10^−180^), taxonomy (Partial *R* ^2^ = 0.25, *F*_(10,2275)_ = 5.32, *P* = 9.91 × 10^−7^), library size (Partial *R* ^2^ = 0.03, *F*_(1,2275)_ = 4.43, *P* = 0.04) and read treatment (Partial *R* ^2^ = 0.01, *F*_(1,2275)_ = 22.96, *P* = 1.77 × 10^−6^). Masurca and SPAdes had a significant positive effect, IDBA had a negative effect and Megahit had no effect on N50 relative to ABySS (Table S4). In contrast to BUSCO%, N50 was affected by read treatment, showing the importance of longer reads in producing less fragmented assemblies.

**Fig. 5:**
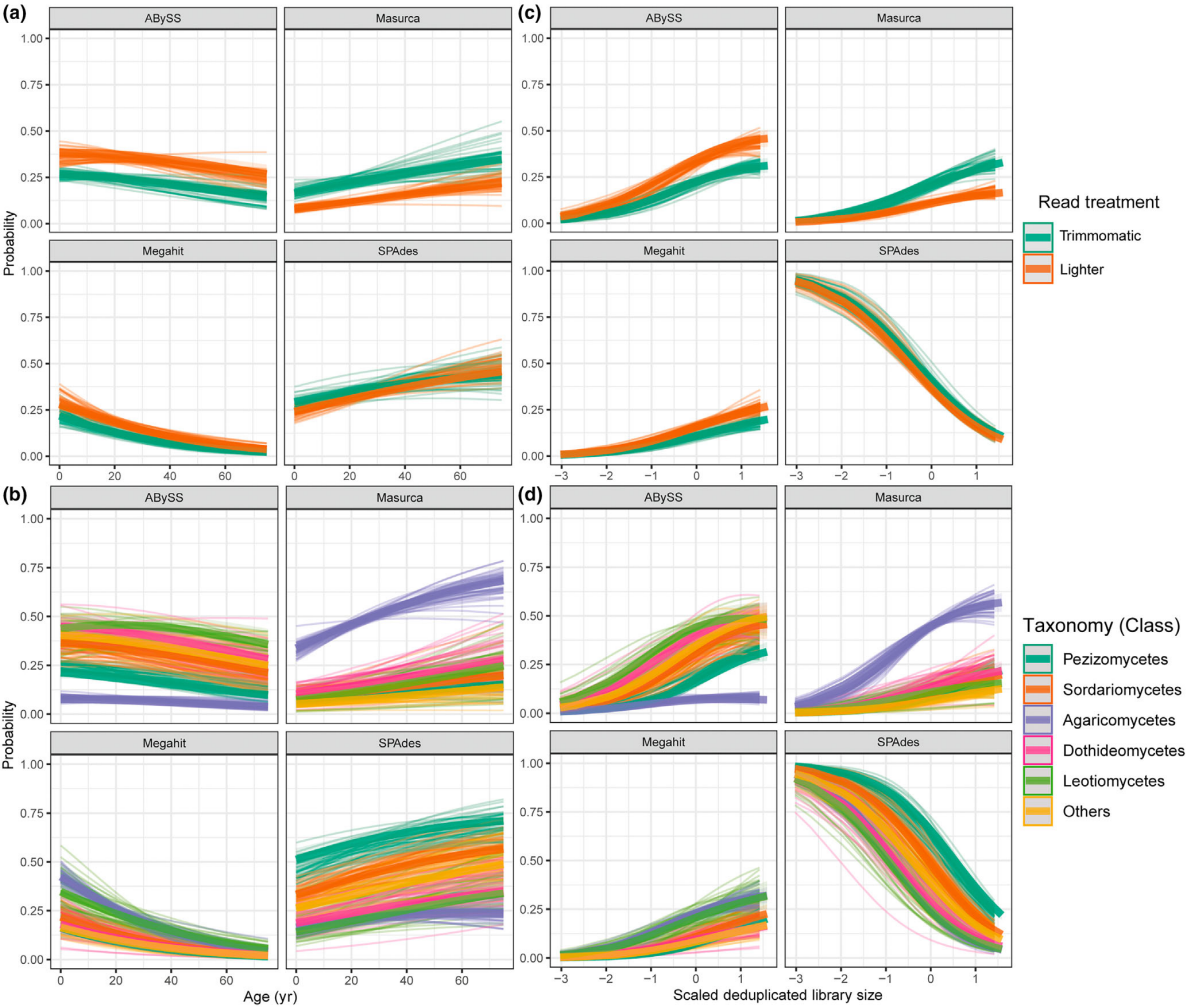
The probability (*y*‐axis) that a genome assembly method gives the best assembly. Note that IDBA was part of the prediction, but it is not depicted due to the close‐to‐zero probabilities. (a) The connection between the specimen's age, the read treatment and the choice of an assembly method. (b) The connection between the specimen's age, the taxonomic identity (classes) and the choice of an assembly method. (c) The connection between the deduplicated library size, the read treatment and the choice of an assembly method. (d) The connection between the deduplicated library size, the taxonomic identity (classes) and the choice of an assembly method.

Given that the choice of assembler showed the biggest effect on genome quality, we assessed whether we could ascertain what conditions may favour an assembler over another. Hence, we first chose the best assembly defined by BUSCO% and performed a multinomial logistic regression (Table S9). After choosing significant predictors via model comparisons, we found that age, read treatment, library size and taxonomic identity were associated with the probability of choosing an assembler (Figs 5, S4), with the exception of IDBA, which was outperformed by other methods in most but eight cases when it produced the same BUSCO% as other methods, yet resulted in more fragmented genomes. A one‐unit increase in the specimen's age is associated with a 0.002 probability increase in the SPAdes performing the best (*P* = 0.036). This suggests that the SPAdes assembler performs better on older specimens than other methods. Yet, if we removed 10 samples that were older than 80 yr to eliminate uncertainties introduced by a low number of records at older ages, a significant increase in the probability of not only SPAdes but Masurca (ME = 0.003, *P* = 0.034) and a decrease in that of Megahit (ME = 0.005, *P* = 0.011) was found (Fig. 5a). Read treatment also caused different outcomes, because Masurca with quality trimming (ME = 0.135, *P* = 0.007) and ABySS with read correction (ME = 0.084, *P* = 0.074) had a higher probability of resulting in the best assembly (Fig. 5a,c).

We also examined the relationship between the deduplicated library size and the probability of an assembly method producing the best assembly. A one‐unit increase in the deduplicated library size was associated with an increase in the probability of choosing Masurca (ME = 0.084, *P* = 0.019) or Megahit (ME = 0.073, *P* = 0.044) and a decrease in that of choosing SPAdes (ME = −0.210, *P* = 3.07 × 10^−7^). The huge drop in the probability of SPAdes with a large library size (Fig. 5c) indicates that SPAdes could outperform other methods if the library size is small, but this advantage disappeared when enough reads were available. Variations across taxonomic groups were also found (Fig. 5b,d). The probability of choosing ABySS (ME = −0.135, *P* = 0.048) or SPAdes (ME = −0.277, *P* = 0.001) was significantly lower for *Agaricomycetes* than for *Pezizomycetes*. At the same time, Masurca outperformed other methods in most *Agaricomycetes* genome assemblies (ME = 0.311, *P* = 3.60 × 10^−6^). All the patterns described above were validated by sampling via fivefold cross‐validation, suggesting that our result was robust and that the sample size was sufficient in this analysis (Fig. 5).

To demonstrate further that genome assembly of historical specimens can be improved by automating multiple assembly approaches, we reanalysed 162 published genomes (Bradshaw *et al*., 2024; Tremble *et al*., 2024) produced from specimens with age ranging from 2 to 82 yr and compared the single‐copy BUSCO% of the published assembly and the best assembly our automated pipeline produced. In all but 13 cases, the automated pipeline resulted in a better genome by increasing the BUSCO% of the assemblies by an average of 21% (Fig. S5). Among these were 35 assemblies where the BUSCO% was increased by more than 40%, highlighting the importance of a multi‐approach bioinformatic analysis of challenging historical specimens.

## Discussion

Historical collections have proved to be essential resources for modern science, helping to better understand crop biology (Gutaker *et al*., 2019), biodiversity and evolution (Dentinger *et al*., 2016; Burbano & Gutaker, 2023; Kuhnhäuser *et al*., 2025). Collections are increasingly being utilised and mined with diverse omics tools, such as high‐throughput amplicon sequencing, metabolomics, bait sequencing and WGS (e.g. Dentinger *et al*., 2016; Forin *et al*., 2018; Funk, 2018; Bradshaw *et al*., 2022; Liimatainen *et al*., 2022). From these methods, bait sequencing has become a standard for plant phylogenetics (Zuntini *et al*., 2024) as it can overcome the limitation of sequencing large‐sized polyploid genomes, generating data that otherwise could not be analysed in conventional phylogenetic studies. Alternatively, WGS could be a viable and cost‐effective option for organisms with small‐ to medium‐sized genomes – such as fungi, which average *c*. 50 Mbp – especially when genomic data are needed for purposes beyond phylogenetics. A few studies have already implemented bait sequencing (Widhelm *et al*., 2019; Liimatainen *et al*., 2022) and WGS using fungal collections (Dentinger *et al*., 2016; Bradshaw *et al*., 2024; Tremble *et al*., 2024; Dirks *et al*., 2025). Yet, no comparative methodological study has been carried out to uncover the full potential of fungariomics.

This study aimed to investigate the factors that affect the success of WGS historical fungal specimens. We assessed four steps: DNA extraction (2524 DNA extractions representing 2104 specimens), barcoding (1686 PCR reactions and 771 sequences), library construction (442 specimens were involved in this step and 392 specimens WGSed) and genome assembly methods (220 libraries were analysed).

We found that the classes *Agaricomycetes* and *Pezizomycetes* had the biggest effect on the DNA yield. Studies have already implied that the success rate of molecular work on historical specimens depends on taxonomy (Osmundson *et al*., 2013; Dal Forno *et al*., 2022). It was indicated that *Basidiomycota* has a higher success rate based on lichen‐forming *Basidiomycetes* data (Dal Forno *et al*., 2022), but we found that class representatives from both *Basidiomycota* and *Ascomycota* had a positive effect on the DNA yield. Because this effect was independent of the input tissue weight and these classes primarily include species with fleshy fruiting bodies and easily accessible spore‐bearing tissue, we suggest that the DNA yield depends on the successful sampling of the spore‐bearing tissue – which varies among taxa – as well as other taxon‐specific features, such as chemical composition (Schrader *et al*., 2012). The higher DNA yield from spore‐bearing tissues has been previously reported, showing that it can be twice the DNA concentration compared to nonspore‐bearing tissues from specimens collected between 1981 and 1990 (Dentinger *et al*., 2010).

We also found a difference in the effect of extraction methods. Our results further support previous studies focused on historical plant specimens that have shown that PTB buffer‐based extraction methods produce a higher total DNA yield than most commonly used commercial kits (Marinček *et al*., 2022) and suggest that PTB buffer‐based extractions can aid in the extraction of the short fragments associated with historical specimens (Gutaker *et al*., 2017). We did not see a significant increase in total DNA yield between CTAB and PTB lysis buffers; instead, our results suggest that a nonsilica‐column‐based purification method may be a more important factor than the lysis buffer in predicting extraction success.

We found a weak positive effect of cold climate on DNA yield. Because climatic classes hold many confounding effects (different species adapted to different environments, preservation methods used, time of collection, etc.), we can only hypothesise about the factors behind this pattern. For example, a colder climate could help with DNA preservation at the stage of specimen collection in the field (Reed *et al*., 2003; Willerslev *et al*., 2004), or fruiting bodies are generally darker in cold temperatures (Krah *et al*., 2019) due to melanin pigments which in turn can provide protection against mechanical and chemical stress (Cordero & Casadevall, 2017). Yet, secondary metabolites do interfere with molecular biology assays; thus, extra caution is needed when interpreting climatic data. Furthermore, melanised fungi can also be found in arid conditions, but at the same time, tropical climates could decrease DNA quality, and studies have suggested that regions with high temperature and humidity are unlikely to provide adequate conditions for aDNA preservation, making the cool‐climate specimens more likely to yield better DNA by comparison (Gutiérrez‐García *et al*., 2014). In contrast to our findings, a meta‐analysis study on herbarium sequencing at the Royal Botanic Gardens, Kew, found little connection between climate and plant DNA yield (Brewer *et al*., 2019). Many studies have successfully barcoded (Brock *et al*., 2009) or even sequenced the whole genomes of more than half‐century‐old fungarium specimens (Staats *et al*., 2013). Similar to our findings, a low correlation was found between the DNA concentration and age of herbaria specimens spanning more than 200 yr (Brewer *et al*., 2019). Despite our efforts, the dataset and models could be improved in the future by including more observations and extra explanatory variables to disentangle the effect of complex variables such as taxonomy and climatic classes. The variables examined in our study explained 27% of the variance in the total DNA; thus, there could be other variables, such as cell density or chemical content, that could further affect DNA yield from historical specimens (Canales *et al*., 2022). Finally, further development of extraction methods for fungarium specimens is needed to improve and extend protocols for high‐throughput projects (e.g. Holmquist *et al*., 2025).

We introduced only 442 extractions in the library construction step due to resource limitations, and chose a library construction method based on the lower limit of input DNA of the commercial kits. The only modification we made was to skip DNA fragmentation if we did not detect high molecular weight fragments using a TapeStation device. Importantly, DIN values were not useful at this step; instead, we manually assessed each electropherogram.

We produced 396 Illumina short‐read libraries with 311–700 mean insert sizes that resulted in high‐quality genomes in many cases. Out of the 220 nonlichen genomes, 159 produced high single‐copy BUSCO% (> 90%) and covered taxonomic orders with a limited number of genomes available, providing useful resources for future phylogenetic and comparative genomic analyses. By developing an automated assembly pipeline utilising 16 different assembly methods, we could conclude some general patterns that could help in future large‐scale sequencing projects. For example, the assembly quality could be increased by sequencing more reads per library. Still, if only a low number of reads were available, the SPAdes assembler had a higher chance of producing a good‐quality assembly (single‐copy BUSCO% > 90). At the same time, reads from older specimens (> 40 yr old) can be assembled best with ABySS and Masurca in half the cases, and SPAdes could provide the best results for the rest of the genomes. We found that applying k‐mer‐based read correction on the libraries can produce less fragmented genomes compared to read quality trimming. Yet, ABySS and Masurca worked best with read correction and quality trimming, respectively. Finally, a variation between taxonomic classes was also found. *Agaricomycetes* could be assembled best with Masurca in around half of the cases, while genomes of *Pezizomycetes* could be assembled better with ABySS or SPAdes among most historical specimens. What genomic features and assembly arguments contribute to the pattern we found is out of the scope of this study; still, it could be the subject of future projects to improve the bioinformatic workflow of diverse taxa. Our study provided valuable *de novo* genome assemblies for future studies and could help with designing upcoming fungarium sequencing research, highlighting the importance of a multi‐method approach in large‐scale historical collection sequencing projects.

## Competing interests

None declared.

## Author contributions

EG, TV and RW conceived the study. TV developed bioinformatic tools, performed the analyses and wrote the first draft of the manuscript. TV, RW, FP and EG designed the experimental work, sampling strategy and analytical framework. RH performed spatial analyses. RW and FP led the molecular work and data management. RH, AYB, TL, EH, BW, JL, JB, QL, KH and RPB conducted molecular work. TV, AYB, BJPS, SLEB, CG, LG, RLM, SLR, MCL, ER, RP‐B and EG processed Sanger sequencing results. RP‐B, TL and EG carried out fieldwork and provided samples. LGN provided additional samples and sequences. AJB facilitated the publication of genomic data. TV, FP and AB assembled the genomes. All authors edited and contributed to the final version of the manuscript. TV, RW and FP contributed equally to this work.

## Disclaimer

The New Phytologist Foundation remains neutral with regard to jurisdictional claims in maps and in any institutional affiliations.

## Supporting information


**Fig. S1** Correlation between weight and total DNA.
**Fig. S2** The relationship between duplicated BUSCO% and specimen collection date.
**Fig. S3** The connection between taxonomy and assembly methods explaining the single‐copy BUSCO%.
**Fig. S4** Multinomial logistic regression‐based estimates of assembly method choice to achieve the best assembly along the specimen's age and two read treatment methods.
**Fig. S5** The comparison of re‐assembled published historical genomes.

**Table S1** The sampled specimens, their metadata and DNA quality measurements.

**Table S2** The main settings and parameters of the automated genome assembly pipeline.

**Table S3** Multivariate linear regression analysis of the DNA yield.

**Table S4** The list of barcoded specimens.

**Table S5** Statistics of the total DNA, DIN, fragment size, library concentration and insert size during the library construction steps.

**Table S6** Metainformation and statistics of assemblies.

**Table S7** The results of the analyses of rDNA extracted from the genomes.

**Table S8** The results of the linear mixed‐effects analyses on the genome assembly methods.

**Table S9** The results of the multinomial logistic regression analyses on choosing the best‐performing assembly method.
Please note: Wiley is not responsible for the content or functionality of any Supporting Information supplied by the authors. Any queries (other than missing material) should be directed to the *New Phytologist* Central Office.

## Data Availability

Barcoding sequences and sequenced libraries were deposited into GenBank (BioProject accession no. PRJNA1279621). Raw genome assemblies are available upon request. The code of the automated pipeline is available on GitHub (https://github.com/vtorda/GenomeAssembler) and in a Docker container (https://hub.docker.com/r/vtorda/assembly_v0.0825).
