# Supplementary material for: Whole genome sequencing of historical specimens from the world's largest fungal collection yields high‐quality assemblies
Source: New Phytol. 2025 Aug 18;251(2):752–67. doi: 10.1111/nph.70472 (PMC13278644; doi:10.1111/nph.70472)

New Phytologist

***New Phytologist* Supporting Information**

Article title: Whole genome sequencing of historical specimens from the world's largest fungal collection yields high-quality assemblies

Authors: Torda Varga*, Roseina Woods*, Frances Pitsillides*, Rowena Hill, Alona Yu. Biketova, Theo Llewellyn, Brandon J.P. Shaw, Emily Hodgson, Brigid Wong, Jasmine Le, Josepha Becker, Alexander J. Bradshaw, Seth L. E. Blake, Clementine Geeves, Quentin Levicky, Lottie Goodman, Ruben L. Mole, Sidney L. Reed, M. Carly Lin, Emily Read, Keenan Harris, Raquel Pino-Bodas, László G. Nagy, Anna Bazzicalupo, Ester Gaya
Article acceptance date: 26 June 2025

The following Supporting Information is available for this article:

Fig. S1 Correlation between weight and total DNA.

Fig. S2 The relationship between duplicated BUSCO% and specimen collection date.

Fig. S3 The connection between taxonomy and assembly methods explaining the single copy BUSCO%.

Fig. S4 Multinomial logistic regression-based estimates of assembly method choice to achieve the best assembly along the specimen's age and two read treatment methods.

Fig. S5 The comparison of re-assembled published historical genomes.

Table S1 The sampled specimens, their metadata and DNA quality measurements.

Table S2 The main settings and parameters of the automated genome assembly pipeline

Table S3 Multivariate linear regression analysis of the DNA yield.

Table S4 The list of barcoded specimens.

Table S5 Statistics of the total DNA, DIN, Fragment size, Library concentration, and insert size during the library construction steps.

Table S6 Metainformation and statistics of assemblies.

Table S7 The results of the analyses of rDNA extracted from the genomes.

Table S8 The results of the linear mixed effect analyses on the genome assembly methods.

Table S9 The results of the multinomial logistic regression analyses on choosing the best-performing assembly method.

Fig. S1 Correlation between weight and total DNA. Pearsons's $R^2 = 0.08$

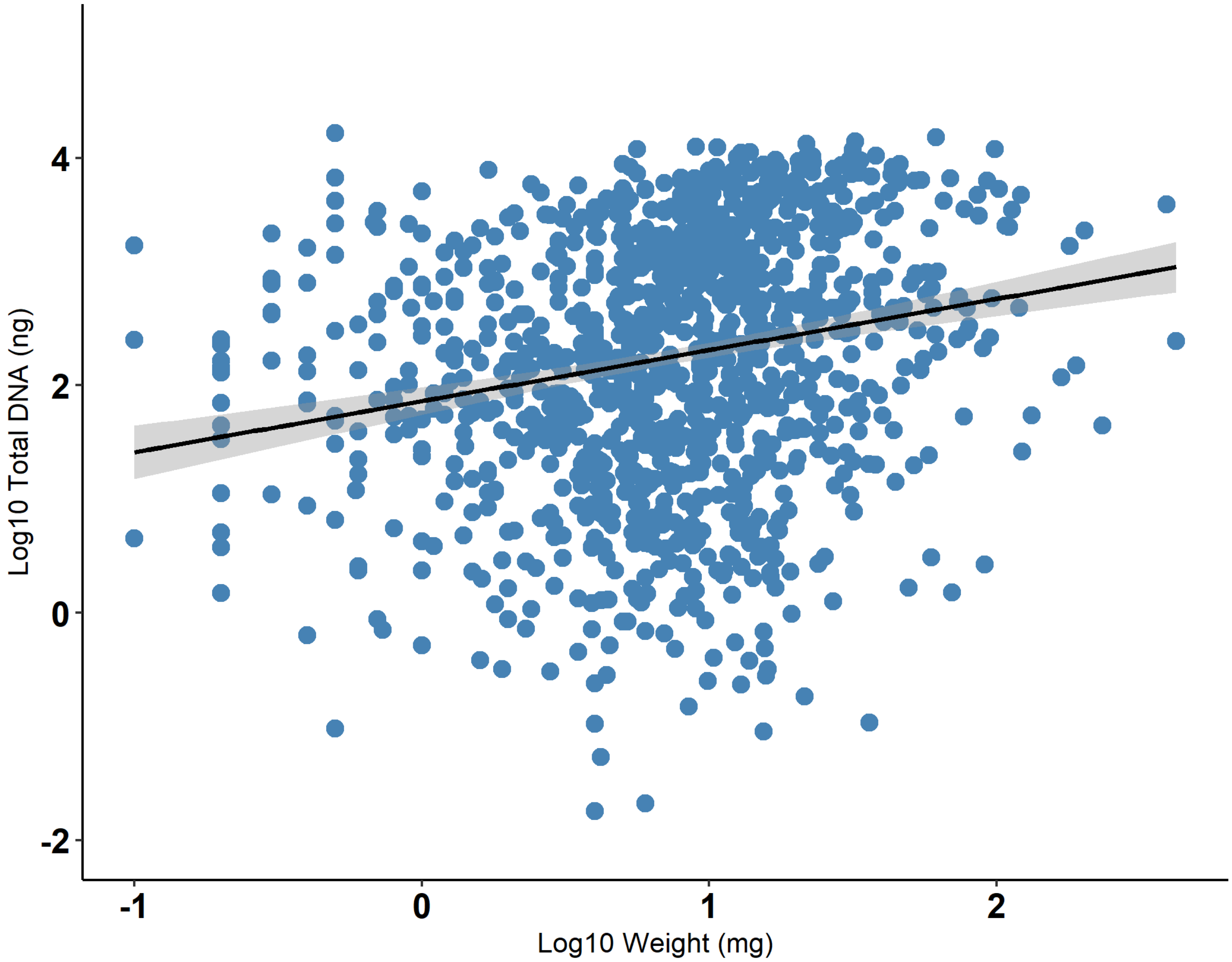

Fig. S2 The relationship between duplicated BUSCO% and the specimen collection date.

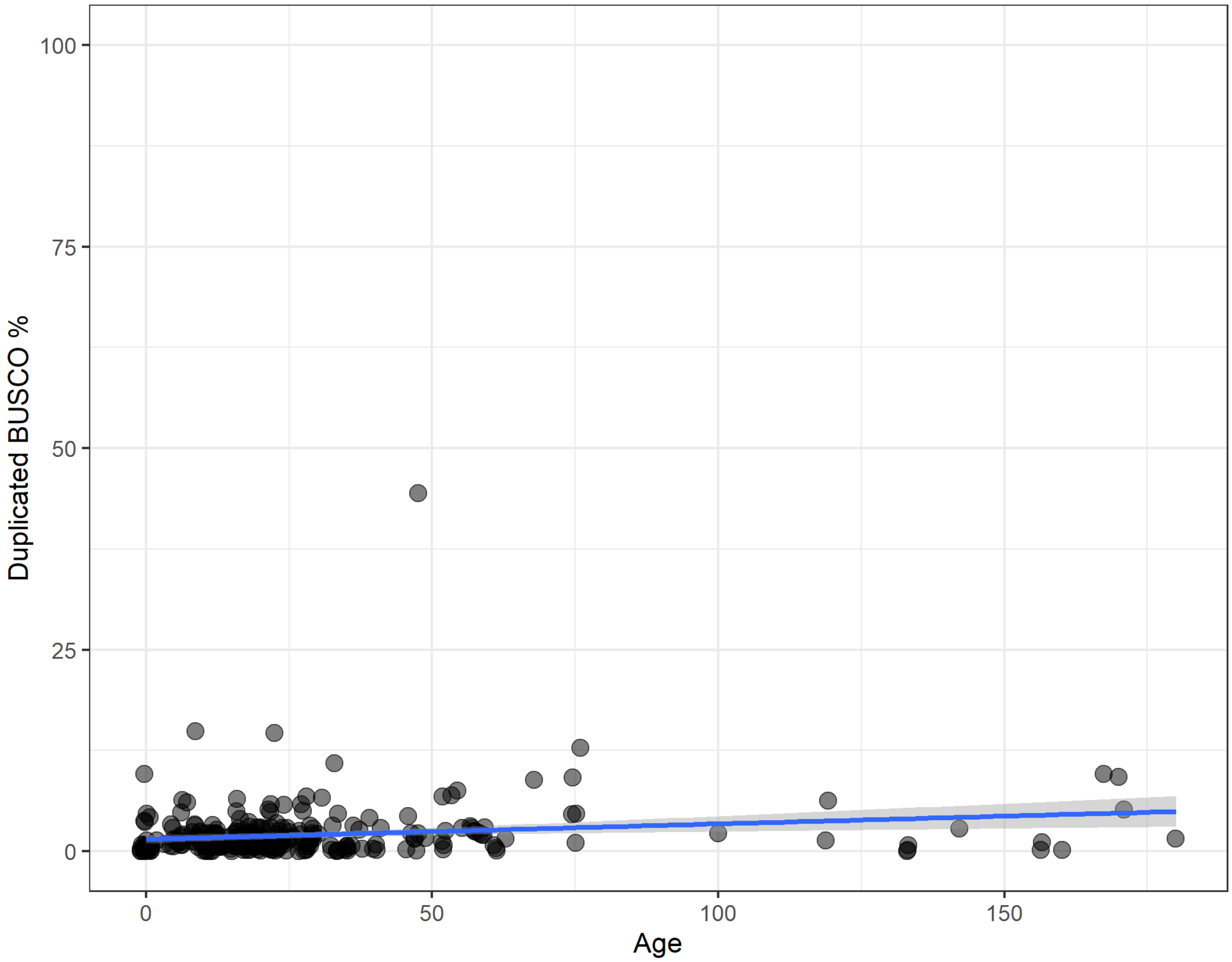

Fig. S3 The connection between taxonomic classes and assembly methods explaining the single copy BUSCO%.

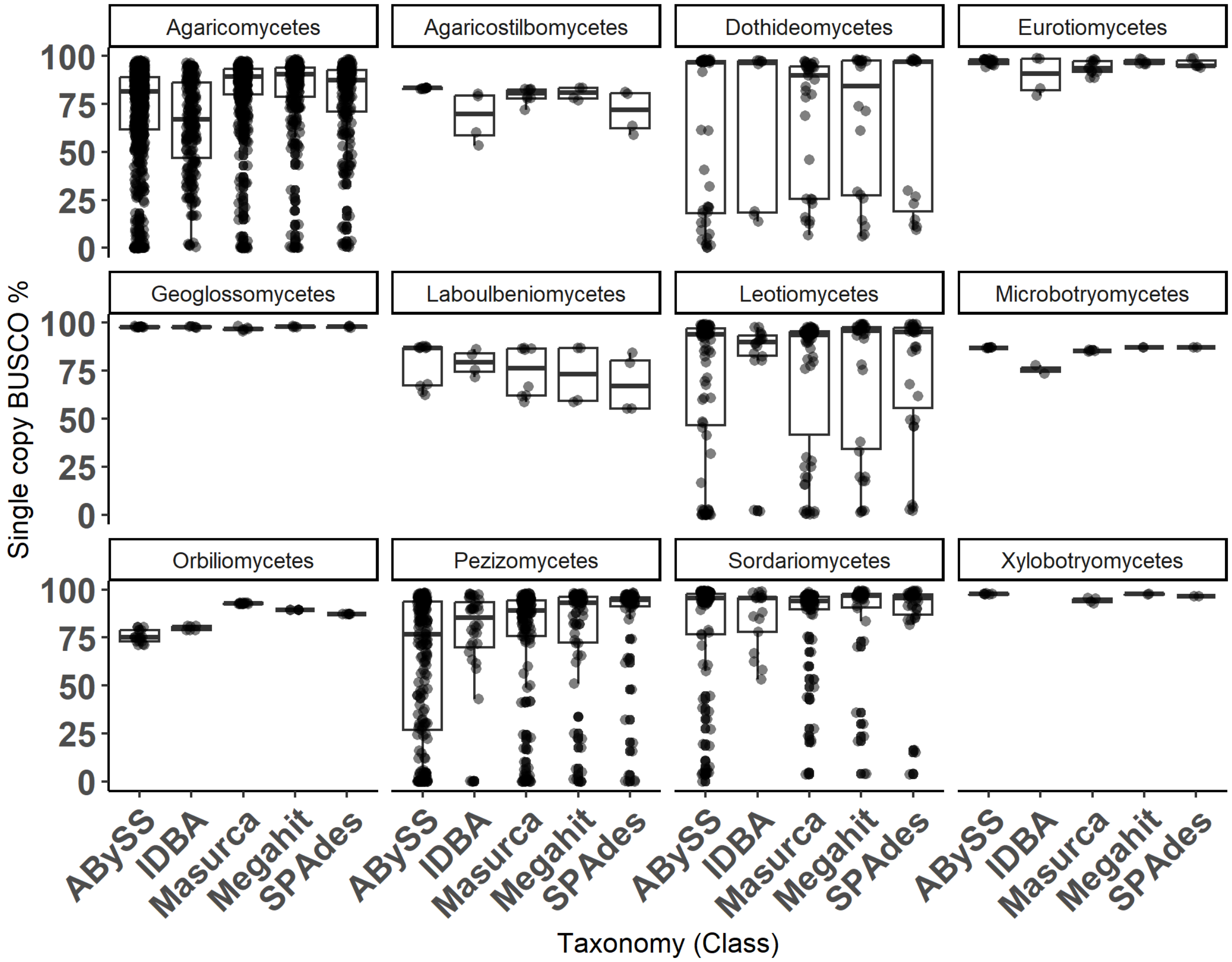

Fig. S4 Multinomial logistic regression-based estimates of assembly method choice to achieve the best assembly along the specimen's age and two read treatment methods. Wide bold lines are the estimates based on the whole dataset, while thin lines are estimates coming from 5-fold cross-validation samples. Note that the age range spanned 161 years compared to Fig. 4 (age < 80 years), because of five specimens that were removed from Fig. 4, yet the main patterns are similar.

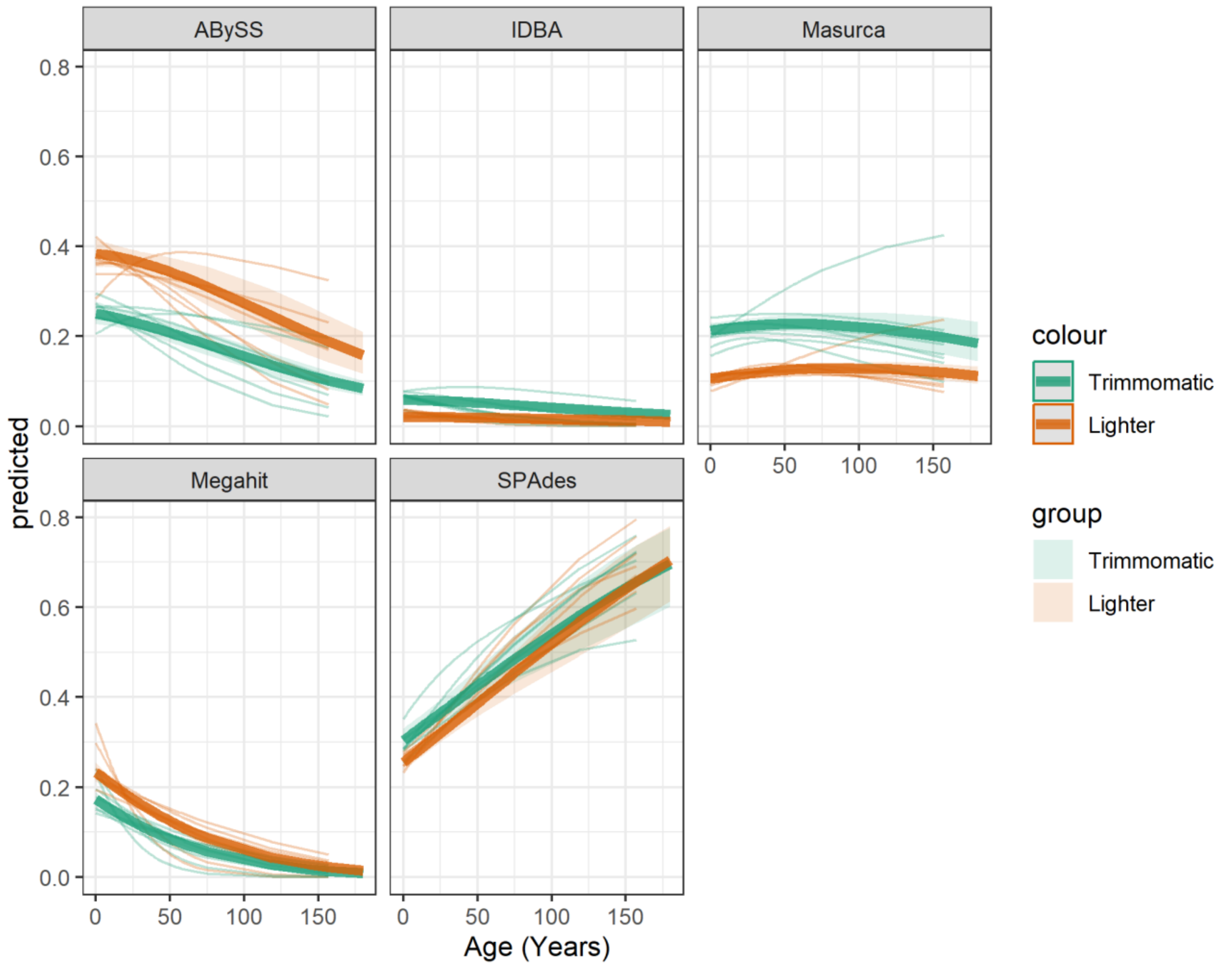

Fig. S5 The comparison of re-assembled published historical genomes. Note that re-assembled (assembled in this study) genomes have a narrower interquartile region, and the BUSCO% was improved in most of the cases (orange lines).

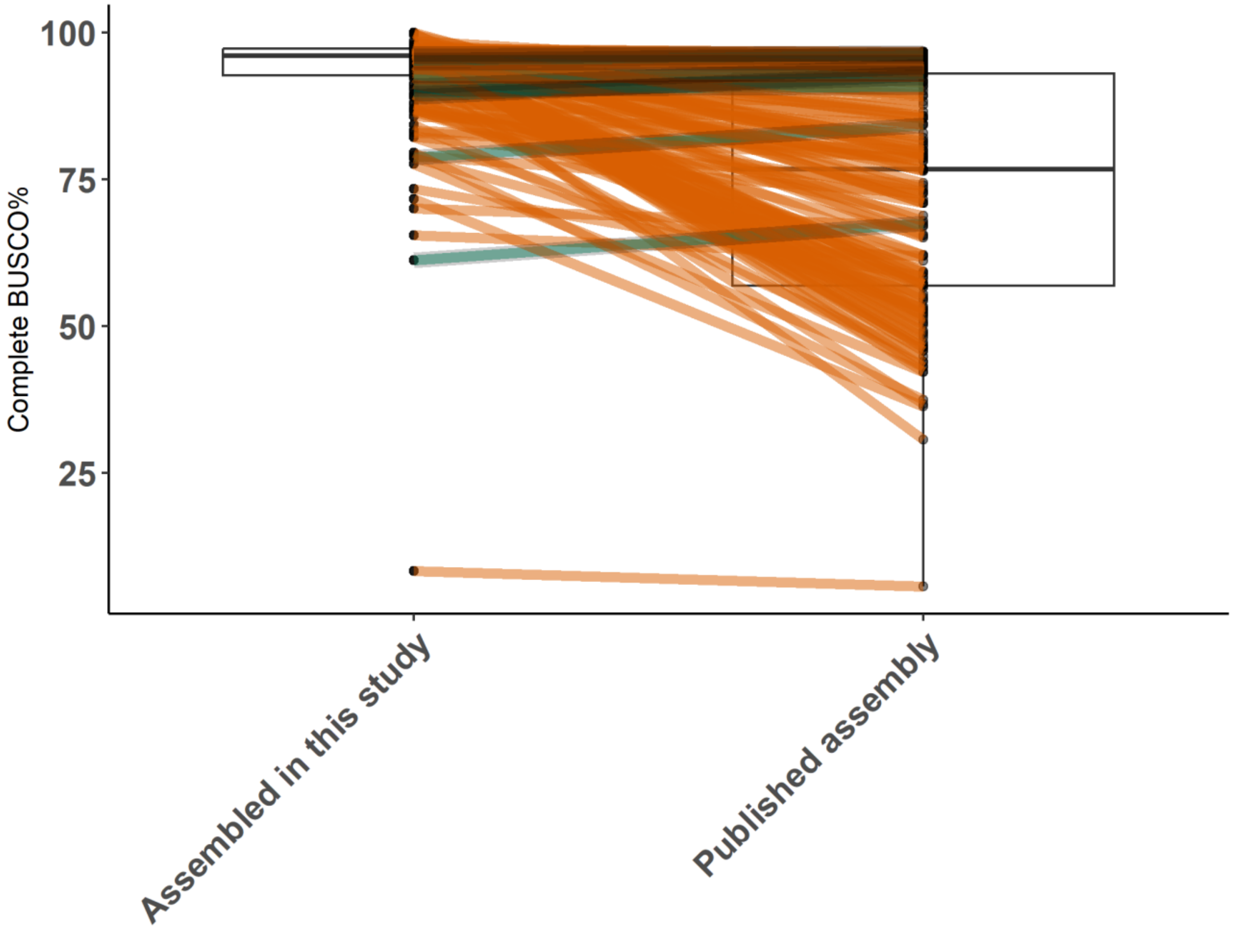

Supplement: Supplementary file 1 — Fig. S1 Correlation between weight and total DNA. Fig. S2 The relationship between duplicated BUSCO% and specimen collection date. Fig. S3 The connection between taxonomy and assembly methods explaining the single‐copy BUSCO%. Fig. S4 Multinomial logistic regression‐based estimates of assembly method choice to achieve the best assembly along the specimen's age and two read treatment methods. Fig. S5 The comparison of re‐assembled published historical genomes. [file NPH-251-752-s005.pdf]
